# Sensorless FOC Performance Improved with On-Line Speed and Rotor Resistance Estimator Based on an Artificial Neural Network for an Induction Motor Drive

**DOI:** 10.3390/s150715311

**Published:** 2015-06-29

**Authors:** Jose M. Gutierrez-Villalobos, Juvenal Rodriguez-Resendiz, Edgar A. Rivas-Araiza, Moisés A. Martínez-Hernández

**Affiliations:** Laboratorio de Mecatrónica, Universidad Autónoma de Querétaro, Cerro de las Campanas, Col. Las Campanas, S/N, Queretaro 76010, Mexico; E-Mails: juvenal@ieee.org (J.R.-R.); erivas@uaq.mx (E.A.R.-A.); moises.martinez@uaq.mx (M.A.M.-H.)

**Keywords:** adaptive system, neural networks, on-line identification, adjustable speed driver, parameter estimation, FOC

## Abstract

Three-phase induction motor drive requires high accuracy in high performance processes in industrial applications. Field oriented control, which is one of the most employed control schemes for induction motors, bases its function on the electrical parameter estimation coming from the motor. These parameters make an electrical machine driver work improperly, since these electrical parameter values change at low speeds, temperature changes, and especially with load and duty changes. The focus of this paper is the real-time and on-line electrical parameters with a CMAC-ADALINE block added in the standard FOC scheme to improve the IM driver performance and endure the driver and the induction motor lifetime. Two kinds of neural network structures are used; one to estimate rotor speed and the other one to estimate rotor resistance of an induction motor.

## 1. Introduction

Three-phase induction motors (IMs) have become the most employed electrical device in industry, thanks to their robustness, efficiency and low maintain. Nowadays, IMs consume 60% of electricity that industry requires, which represents the 21% of global electrical production, about 3.99 PetaWatts-hour [1]. Therefore, in order to increase the IM efficiency and raise its performance, any improvement results in a global benefit and helps the industrial economy. Current drivers are based on control schemes such as voltage-frequency (V/F), direct torque control (DTC) and field oriented control (FOC). FOC has been the most selected control method for IMs in torque and speed control applications where high performance is required. There have been many contributions towards improve it for almost 3 decades [2]. Basically, FOC sees the IM as a direct current (DC) motor and it has been classified in two types. One method has been the direct field oriented control (DFOC), which needs to know the position of the field magnetic flux and the rotor magnetic flux with hall-effect sensors inside the electric machine, which results complicated or impossible to installation of the sensors. The other scheme is indirect field oriented control (IFCO), which has two different diagrams. The sensored diagram estimates magnetic flux position and uses an encoder to measure the IM rotor speed. The sensorless scheme estimates magnetic flux as well but it does not need an encoder to know the rotor speed. Sensorless IFCO estimates the motor velocity based on the number of IM poles, and the frequency of voltages and currents applied to stator coils [3]. Currently, commercial drivers use sensorless IFCO because it does not need an encoder. It is to say, it is a simple controller with good performance and acceptable results for some applications.

**Figure 1:**
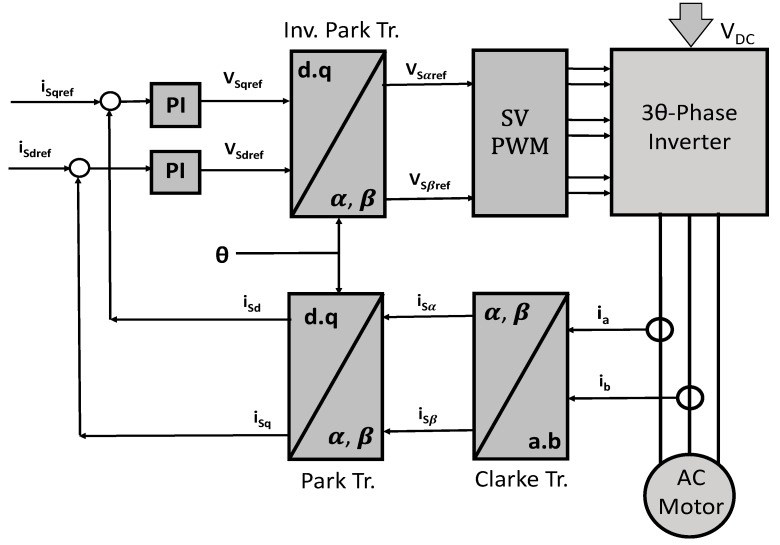
General sensorless indirect field oriented control (IFCO) scheme.

As explained before, IFOC is better known only as FOC in the IM driver and controller literature. It is the control technique that has gained more interest within researchers because there have been opportunity areas for control theory, observers and estimators. Figure 1 shows the general sensorless FCO scheme. For the reason of not using sensors and estimating field magnetic flux, there have been many surveys in this area. Other, previously published reviews such as [4] analyze and describe several methods, where observers, estimators and controllers were used. Never the less, there are some works that only presented validations for short times such as [5,6,7], which do not really ensure that their systems work properly, after longer terms or under motor condition variations.

This work employs a combination of two different artificial neural network (ANNs) approaches. The combination of tow neural networks (NNs) helps to improve IM-driver performance, by means of reducing the mismatch produced between estimated electrical parameters values and the real ones. The ANN unit estimates motor speed (ω_*r*_) and rotor resistance (*R_r_*), which allows us to keep the IM controller tuned, achieving higher control over a wide speed range and load variations. Since *R_r_* varies by up to 100% from its original value due to temperature changes and load steps [8]. Normal control schemes can present undesirable speed tracking at low speeds. It leads to decreases in IM efficiency and shortens motor drive life.

**Figure 2:**
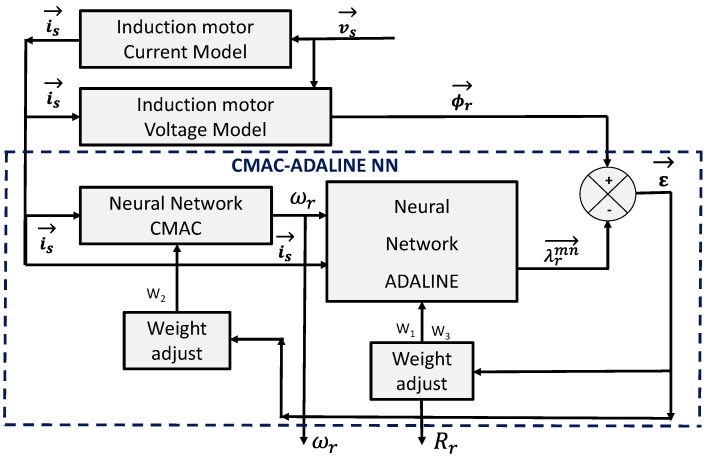
Cerebellar model articulation controller (CMAC)-adaptive linear neuron (ADALINE) structure for the parameter estimator.

In this algorithm, a cerebellar model articulation controller (CMAC) was used to estimate ω_*r*_, it is selected for its capability of quickly learning non-linear functions due to the local nature of its weight modification. These networks are simple to implement when compared to other types of neural network as describe in [9]. Then, for *R_r_* estimation, a block with an adaptive linear neuron (ADALINE) structure is selected. The main advantages of this ANN are its simplicity and the ability to be trained online; besides, the ADALINE weights can be interpreted physically, as explained in [10]. Figure 2 presents the proposed ANN scheme.

This survey is divided into four sections. The first part, which describes how this algorithm was implemented and its outcomes, the second part presents the ANN structures employed in the research and their behavior, the third part explains how CMAC-ADALINE unit performs on-line parameter estimation and improves drive speed control performance, the forth part shows conclusive results and the final section contains the conclusions and the possible future applications for this work.

### 1.1. CMAC Description and Behavior

**Figure 3:**
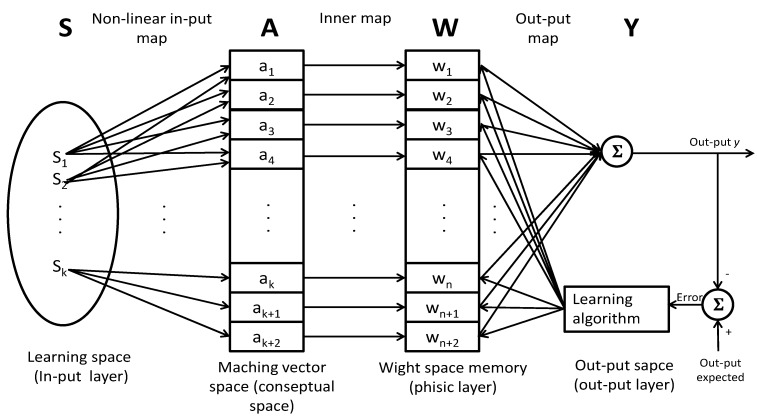
Artificial neural network (ANN) CMAC model.

The CMAC structure was developed (such as, the human cerebellum), and it is a convenient learning structure for on-line and real-time modeling and controlling induction motors, IMs are non-linear systems. CMAC is capable of learning non-linear functions; it can be defined as a look-up-table. In this structure, each state variable is quantized and mapped, see Figure 3. The space vector is divided into discrete states. The output of each state can be obtained from the sum of the information stored in the associate memories.

The state vector is mapped in *S^th^* elements onto another vector called *A*, which is formed by *n^th^* elements. In the next map, the output *P* is the sum of the weights whose activation function will be activated. Its representative mathematical equations is given as follows:

$$\begin{array}{l} f:s\to A\\ g:A\overset{\;W\;}{\to }P \end{array}$$

Now *s* is an input vector of *k*-dimension, *A* is the *N*-dimension association vector containing *M* active elements, *P* is the output vector and *W* is the corresponding weight vector in which input data of *P* is stored, as explained in [11].

(1) $$P_{k}=g\left(a_{k}^{*}\right)=A_{k}W_{k}$$

In this equation, *k* is associated to *k_th_* states, $a_{k}^{*}$ means the activation function of the *k_th_* state, and *A_k_* is the grouping of cell vector of *k_th_* state. The form CMAC weights are adapted is given by:

(2) $$W_{k}\left(j+1\right)=W_{k}\left(j\right)+\Delta W_{k}\left(j\right)=W_{k}\left(j\right)+\frac{\alpha }{M}\left(Y_{d,k}-P_{k}\left(j\right)\right)$$

(3) $$W_{k}\left(j+1\right)=W_{k}\left(j\right)+\frac{\alpha }{M}\left(Y_{d,k}-A_{k}W_{k}\left(j\right)\right)$$

where *W_k_* means the weight vector at *k*-th state, *j* means *j*-th iteration, α is the learning rate is selected, *M* is the number of activated association cells, and *Y_d,k_* is the desired output at *k*-th state. The learning rule of CMAC is to distribute the error equally to the corresponding weights, as well explained in [11]. In the learning process, the input of the controlled plant U (induction motor) is treated as a desired output to modify the contents of CMAC stored at location *Y*(*k*) and *Y*(*k* + 1), where *Y*(*k* + 1) is the actual system output at time step *k* + 1. How precisely the CMAC can approximate a controlled plant is mainly determined by the quantization in each dimension of the input vector and the generalization width.

### 1.2. ADALINE Description and Behavior

**Figure 4:**
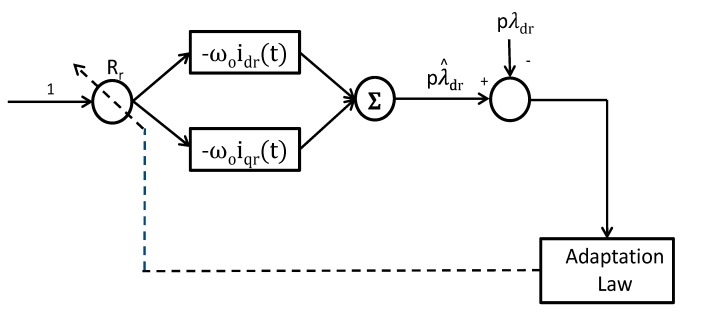
ANN ADALINE model.

In this section, The ADALINE structure is explained in [10]. The ANN-ADALINE structure is equivalent to one neuron which is composed of an input vector *X_k_*, a weight matrix *W_k_*, and an activation function *f*(*v*). The weights vector *W_k_* = [*W_0k_,W_1k_* … *W_nk_*]^*T*^ corresponds to the whole neuron synaptic forces. The input vector *X_k_* = [*X_0k,_ X_1k_* … *X_nk_*] corresponds to the whole neuron input stimulus. The activation function *f*(*v*) specifies the neuron behavior. Various activation functions can be used in the ANN theory. However, the ADALINE uses the linear activation function *f*(*v*) = *v* Consequently, the ADALINE output *y_k_* is given by: *y_k_* = *X_k_W_k_*. When the ADALINE is excited, it produces the output *y_k_* which depends on the input vector. The basic scheme of an ADALINE structure is shown in Figure 4. The weights vector *W_k_* is continuously modified during the network learning process with the purpose of approaching as close as possible the desired output *d_k_*.

It is a linear combination of its in-put vector *X*(*k*) = [*X_1_, X_2_…X_n_*]^*T*^ at any time *k^th^* that is multiplied by the weight vector *W*(*k*) = [*W_1_, W_2_…W_n_*], finally, their products are summed to obtain a linear output represented by:

(4) $$\overset{\wedge }{Y}\left(k\right)=X{\left(k\right)}^{T}W\left(k\right)$$

And adaptation rule is based on the Least-Square-Method (LMS), which is known as the Widro-Hoff delta rule and is given by:

(5) $$\overset{\wedge }{Y}\left(k\right)=W\left(k\right)+\alpha \frac{X\left(k\right)e\left(k\right)}{X{\left(k\right)}^{T}X\left(k\right)}$$

The main drawback with the ADALINE structure consists in selecting the correct set of weights so that the input-output ADALINE behavior gets closer to the desired input-output data. In order to update ADALINE weights, the LMS algorithm was used to minimize the error *e*(*k*). The learning process for ADALINE is well explained in [12]. This structure requires supervised learning. The error is obtained by using the energy function E = (1/2)*e^2^*(*k*) and is calculated by:

(6) $$e\left(k\right)=d_{k}-y_{k}=\;d_{k}-X_{k}^{T}W_{k}$$

The stepping method is described by the expression:

(7) $$W_{k+1}=W_{k}+\eta \left(-{\bar{\nabla }}_{k}\right)=W_{k}-\eta \frac{\partial e_{k}^{2}}{\partial W_{k}}$$

where η is used to control the stability and the convergence rate; *∇_k_* is the gradient values at *W* = *W_k_*.

Differentiating the equation above and introducing the linear error we obtain:

(8) $$W_{k+1}=W_{k}-\eta \frac{\partial e_{k}^{2}}{\partial W_{k}}=W_{k}-2\eta e_{k}\frac{\partial e_{k}}{\partial W_{k}}$$

and

(9) $$W_{k+1}=W_{k}-2e_{k}\eta \frac{\partial (d_{k-}X_{k}^{T}W_{k})}{\partial W_{k}}$$

which results in

(10) $$W_{k+1}=W_{k}-2\eta e_{k}X_{k}^{T}$$

Finally, the constant η determines stability and convergence rate, this value is typically less than one. The training coefficient η is used to accelerate the algorithm convergence and it has to be variable. It has to be high at the beginning of the training process; then, it has to decrease to present a small value at the end. The value of η can be selected by the expression Equation (11) as well explain in [13].

(11) $$\eta =\eta_{i}{\left(\frac{\eta_{f}}{\eta_{i}}\right)}^{\frac{t}{t_{\text{max}}}}$$

where η_*i*_ is the initial value of the learning rate and η_*f*_ is the final value, and *t_max_* is the maximum training time. Never the less, this parameter has to be first obtained by simulation, then the ANN response has to be analyzed to determine the correct value for the physical system.

## 2. Speed and Rotor Resistance Estimator Implementation

Thus, it is important to consider the state-variable model of the induction machines referred to an arbitrarily rotating reference frame [11]. Which is written as

(12) $$\frac{d}{dt}\;\left[\begin{array}{c} i_{S_{d}}^{c}\\ i_{S_{q}}^{c}\\ \psi_{R_{d}}^{c}\\ \psi_{R_{q}}^{c} \end{array}\right]=\left[\begin{array}{cccc} -\frac{R_{S}}{L_{S^{\sigma }}}-\frac{R_{S}(1-\sigma )}{L_{S^{\sigma }}} & \omega_{S} & \frac{{MR}_{S}}{{\sigma L}_{R}L_{R}^{2}} & \frac{{M\omega }_{R}}{{\sigma L}_{R}L_{R}}\\ -\omega_{S} & -\frac{R_{S}}{L_{S^{\sigma }}}-\frac{R_{S}(1-\sigma )}{L_{S^{\sigma }}} & \frac{{M\omega }_{R}}{{\sigma L}_{R}L_{R}} & \frac{{MR}_{S}}{{\sigma L}_{R}L_{R}^{2}}\\ \frac{{MR}_{S}}{L_{R}} & 0 & -\frac{R_{R}}{L_{R}} & \omega_{S}-\omega_{R}\\ 0 & \frac{{MR}_{S}}{L_{R}} & -(\omega_{S}-\omega_{R}) & -\frac{R_{r}}{L_{R}} \end{array}\right]\left[\begin{array}{c} i_{S_{d}}^{c}\\ i_{S_{q}}^{c}\\ \psi_{R_{d}}^{c}\\ \psi_{R_{q}}^{c} \end{array}\right]+\left[\begin{array}{cc} \frac{1}{L_{S^{\sigma }}} & 0\\ 0 & \frac{1}{L_{S^{\sigma }}}\\ 0 & 0\\ 0 & 0 \end{array}\right]\left[\begin{array}{c} \nu_{S_{d}}^{c}\\ \nu_{S_{d}}^{c} \end{array}\right]$$

Besides, this mathematical model is described in [14]. For being able to implement this combined ANNs, it is necessary to consider the IM current model given by Equation (13) and the IM voltage model given by Equation (14). The Equation (14) is required to adjust the weights, and these equations are shown in the following lines.

(13) $$\left[\begin{array}{c} \frac{d\lambda_{dr}^{vm}}{dt}\\ \frac{d\lambda_{qr}^{vm}}{dt} \end{array}\right]=\frac{L_{r}}{L_{m}}\left\{\left[\begin{array}{c} v_{ds}\\ v_{qs} \end{array}\right]-R_{s}\left[\begin{array}{c} i_{ds}\\ i_{qs} \end{array}\right]-\sigma L_{s}\left[\begin{array}{c} \frac{di_{ds}}{dt}\\ \frac{di_{qs}}{dt} \end{array}\right]\right\}$$

(14) $$\left[\begin{array}{c} \frac{d\lambda_{dr}^{im}}{dt}\\ \frac{d\lambda_{qr}^{im}}{dt} \end{array}\right]=\left[\begin{array}{cc} -\frac{1}{T_{r}} & -\omega_{r}\\ \omega_{r} & -\frac{1}{T_{r}} \end{array}\right]\left[\begin{array}{c} \lambda_{dr}^{im}\\ \lambda_{qr}^{im} \end{array}\right]+\frac{L_{m}}{T_{r}}\left[\begin{array}{c} i_{ds}\\ i_{qs} \end{array}\right]$$

The current model of Equation (14) can also be rewritten as:

(15) $$\vec{\lambda_{r}^{im}}=\left(\frac{-1}{T_{r}}I+\omega_{r}J\right)\vec{\lambda_{r}^{im}}+\frac{L_{m}}{T_{r}}\vec{i_{s}}$$

where

$$I=\left[\begin{array}{cc} 1 & 0\\ 0 & 1 \end{array}\right],\;\;\;\;\mathrm{J=}\left[\begin{array}{cc} 0 & -1\\ 1 & 0 \end{array}\right],\;\;\;\;\vec{i_{s}}=\left[\begin{array}{c} i_{\text{ds}}\\ i_{\text{qs}} \end{array}\right]$$

$$\vec{v_{s}}=\left[\begin{array}{c} v_{ds}\\ v_{qs} \end{array}\right],\;\;\;\;\vec{\lambda_{r}^{\text{im}}}=\left[\begin{array}{c} \lambda_{\text{dr}}^{\text{im}}\\ \lambda_{\text{qr}}^{\text{im}} \end{array}\right],\;\;\;\;\vec{\lambda_{r}^{\text{vm}}}=\left[\begin{array}{c} \lambda_{\text{dr}}^{\text{vm}}\\ \lambda_{\text{qr}}^{\text{vm}} \end{array}\right]$$

The discrete data model of Equation (15) can also be expressed as:

(16) $$\vec{\lambda_{r}^{nm}}\left(k\right)=\left(W_{1}I+W_{2}J\right)\vec{\lambda_{r}^{nm}}\left(k-1\right)+W_{3}\vec{i_{s}}\left(k-1\right)$$

Considering

$$W_{1}=1-\frac{T_{s}}{T_{r}};\;\;\;\;W_{2}=\omega_{r}T_{s};\;\;\;\;W_{3}=\frac{L_{m}}{T_{r}}T_{s}$$

and *T*_s_ is the sampling period. Equation (16) can also be written as:

(17) $$\vec{\lambda_{r}^{nm}}\left(k\right)=W_{1}X_{1}+W_{2}X_{2}+W_{3}X_{3}$$

Therefore

$$X_{1}=I\vec{\lambda_{r}^{nm}}\left(k-1\right)=\left[\begin{array}{c} \lambda_{dr}^{nm}\left(k-1\right)\\ \lambda_{qr}^{nm}\left(k-1\right) \end{array}\right]$$

$$X_{2}=J\vec{\lambda_{r}^{nm}}\left(k-1\right)=\left[\begin{array}{c} -\lambda_{dr}^{nm}\left(k-1\right)\\ \lambda_{qr}^{nm}\left(k-1\right) \end{array}\right]$$

$$X_{3}=I\vec{i_{s}}\left(k-1\right)=\left[\begin{array}{c} i_{ds}\left(k-1\right)\\ i_{qs}\left(k-1\right) \end{array}\right]$$

Then the weight adjustment is obtained by trying to minimize the error

(18) $$\vec{\varepsilon }\left(k\right)=\vec{\phi_{r}}\left(k\right)-\vec{\lambda_{r}^{nm}}\left(k\right)$$

The weights between neurons are tuned by minimizing the energy function

(19) $$E=\frac{1}{2}{\vec{\varepsilon }}^{2}\left(k\right)=\frac{1}{2}{\left\{\vec{\phi_{r}}\left(k\right)-\vec{\lambda_{r}^{nm}}\left(k\right)\right\}}^{2}$$

The weight variation is given by

(20) $$\Delta W_{n}\left(k\right)\propto -\frac{\partial E}{\partial W_{n}}=-\frac{\partial E}{\partial \vec{\lambda_{r}^{nm}}\left(k\right)}\frac{\partial \vec{\lambda_{r}^{nm}}\left(k\right)}{\partial W_{n}}=-\vec{\delta }X_{n}$$

where

(21) $$\vec{\delta }=\frac{\partial E}{\partial \vec{\lambda_{r}^{nm}}\left(k\right)}={\left[\vec{\lambda_{r}^{im}}\left(k\right)-\vec{\lambda_{r}^{nm}}\left(k\right)\right]}^{T}$$

Another coefficient (α) is added to determine the effect of the changes of the past weights and increase in the learning rate. Finally, weight adjustment of *W_1_* and *W_3_* can be represented by the next form [15]:

(22) $$W_{1}\left(k\right)=W_{1}\left(k-1\right)-\eta \vec{\delta }X_{1}+\alpha \Delta W_{1}\left(k-1\right)$$

(23) $$W_{3}\left(k\right)=W_{3}\left(k-1\right)-\eta \vec{\delta }X_{3}+\alpha \Delta W_{3}\left(k-1\right)$$

The Equation (11) was used to select a proper value for η, several values had to be tested in order to determine what value is the most convenient.

and considering *W_2_*

(24) $$\Delta W_{2}\left(k\right)={\left(\vec{\phi_{r}}\left(k\right)-\vec{\lambda_{r}^{nm}}\left(k\right)\right)}^{T}J\vec{\lambda_{r}^{nm}}\left(k-1\right)$$

Then the weight *W_2_* value is calculated as follows:

(25) $$W_{2}\left(k\right)=W_{2}\left(k-1\right)+\frac{\alpha }{M}\Delta W_{2}\left(k\right)$$

By using the Equation (26) and solving the speed from the weight *W*_2_, the estimated rotor speed is given by combining equations above, the estimated rotor speed is represented as follows:

(26) $$\omega_{r}(k)=\omega_{r}(k-1)+\frac{\alpha }{M}\frac{\Delta W_{2}(k)}{T_{S}}$$

and rotor resistance can be found from either *W_1_* or *W*_3_ using Equations (27) or (28).

(27) $$R_{r}\;=\frac{L_{r}W_{3}}{L_{m}T_{s}}$$

(28) $$R_{r}\;=\frac{L_{r}\left(1-W_{1}\right)}{T_{s}}$$

**Figure 5:**
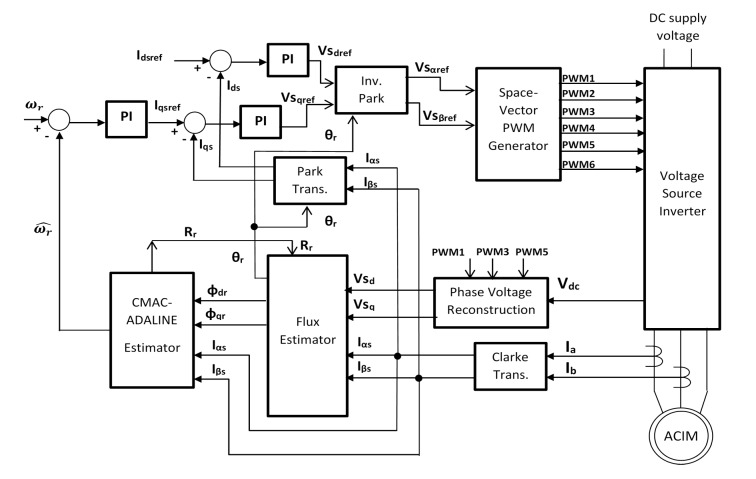
CMAC-ADALINE block connected to the FCO scheme.

Both equations above were tested, but finally Equation (28) was eventually selected to be used because it presented less variation and better response due to it was only affected by *L_r_*. It is important to know how the proposed algorithm is exactly incorporated to the general FCO scheme as shown in Figure 5.

## 3. Experimental Results

This part presents the results obtained from a comparative between standard FOC scheme and the FOC one including the proposed estimating algorithm. The initial values were η = 0.05 and α = 0.01, *T_s_* = 0.001s, this block was implemented and validated in a real-time embedded system TI tool set TMDSHVMTRPFCKIT. Table 1 shows the parameter of the IM.

**Table 1:** Parameters of the induction motor.

1/4 HP, 1725 R.P.M., 208–230 V, 1.2–1.3 A, 3 Phase, 4 Pole, 60 Hz
Stator Resistance *R_s_*: 11.05 Ω
Stator Inductance *L_s_*: 0.316423 H
Rotor Resistance *R_r_*: 6.11 Ω
Rotor Inductance *L_r_*: 0.316423 H
Mutual Inductance *L_m_*: 0.293939 H

The IM is shaft-to-shaft connected to a DC motor, which simulates a changing load. That load can be set or adjusted to validate control performance at different speeds and loads. Estimated ω_*r*_ and *R_r_* are real-time calculated and they get adjusted at every millisecond in the Code Compose control panel included in the TI software. This is suitable to track IM speed, store and validate real and estimated parameter values. The DC motor is controlled with a servo amplifier that has an inner current control loop to drive torque applied to the IM. DC motor features are described in Table 2. 

**Table 2:** Direct current motor parameters.

Power: 1/4 Hp
R.P.M.: 1725
Voltage (dc): 180 V
Current: 2.5 A
Torque constant (Kt): 1 N·m/A
Torque Max: 2.5 N

**Figure 6:**
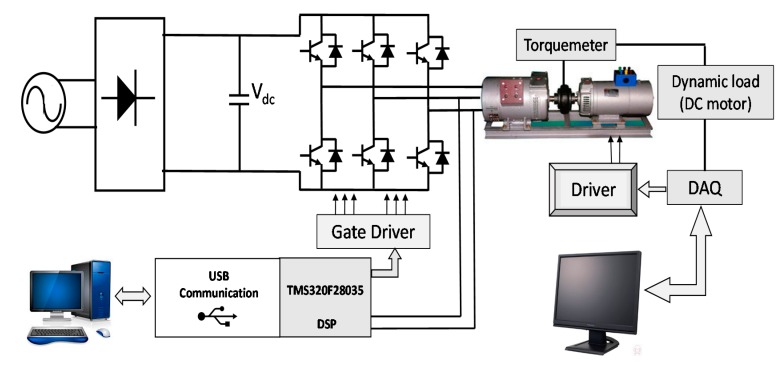
Complete system scheme used to test and validate the algorithm and testbench to simulate different load resistances.

The servo amplifier is connected to a National Instruments (NI) data acquisition board (DAQ) by means of an USB connection, then a human-machine interface (HMI) in LabVIEW is implemented to monitor and control variable torque applied to the AC motor. A load form 0 to 2.5 N·m can be emulated by this system. A FUTEK shaft-to-shaft rotary torque sensor (TRS300) is used to measure the mechanical torque applied to the AC motor, and its signal is connected to an NI DAQ to create the load control system as shown in Figure 6. There were three speeds applied: low speed (LS) at 10% of nominal RPM value, medium speed (MS) at 50% and high speed (HS) at 90%.

**Figure 7:**
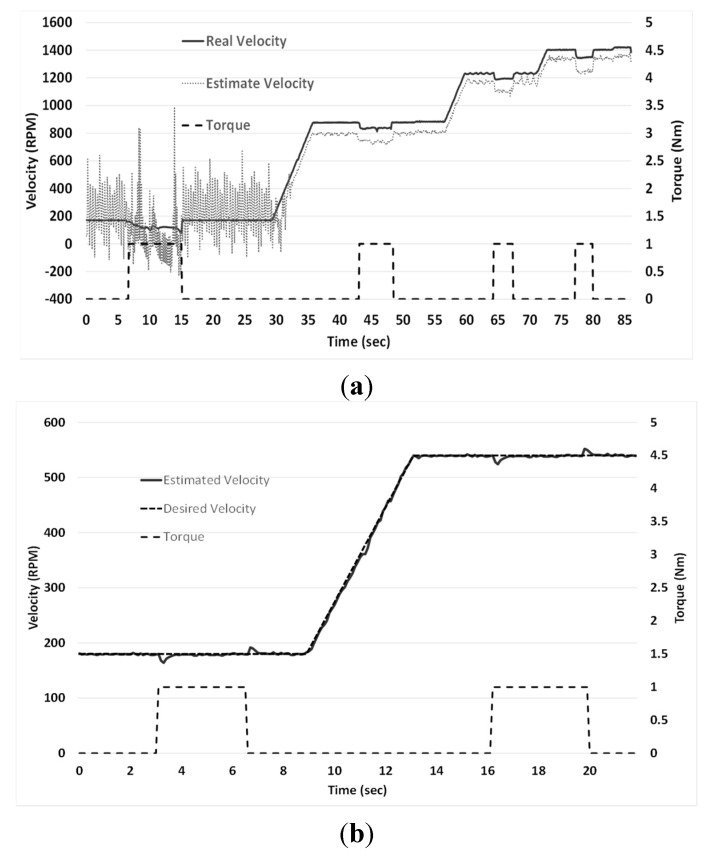
(**a**) FOC without ANN estimator response and (**b**) FOC with the ANN-block.

Sensorless FOC without estimator algorithm was first tested with a specific velocity profile, which consists of speed steps applied at 180, 540, 860, 1260 and 1440 R.P.M. at 1 N·m torque, generated with the DC motor. This created disturbances that directly affected IM speed. Those effects decreased or increased when load was applied or removed. Figure 7a shows the error between the estimated velocity and the real one at low revolutions, however, as speed increases, estimated velocity gets closer to the real value. Figure 7b shows the estimated speed and the desire one with load applied, which consists of speed steps applied at 180 and 540 R.P.M. at 1 N·m torque, using the ANN block, introduced to the standard FOC. The real velocity was measured with a QD200 encoder connected directly to the IM shaft.

**Figure 8:**
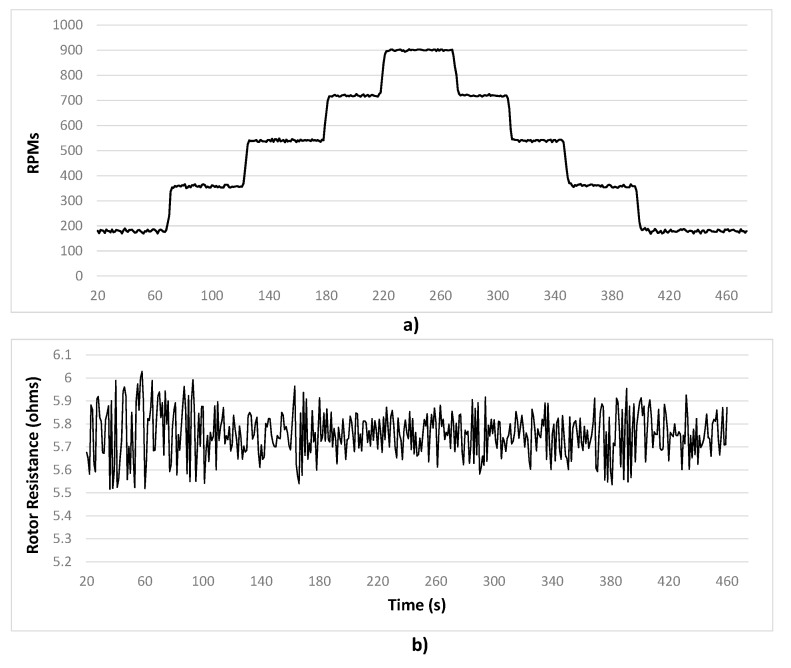
(**a**) Speed changing profile from 180 to 900 R.P.M with 1 N·m. load and (**b**) Comparison between manufacturer motor resistance and estimated one.

Thus, *R_r_* tends to vary within a range close to the manufacturer value, approximately 6.11 Ohms. These changes precisely occur at speed variations. These mismatches affect the controller performance because, as it was described in Section 3, *R_r_* is part of the IM mathematical model that controller uses. Figure 8a shows the speed steps applied to the IM and Figure 8b presents the *R_r_* is estimated with the other ADALINE structure. These changeable values are the ones that are fed back to the controller scheme in order to keep it tuned.

**Figure 9:**
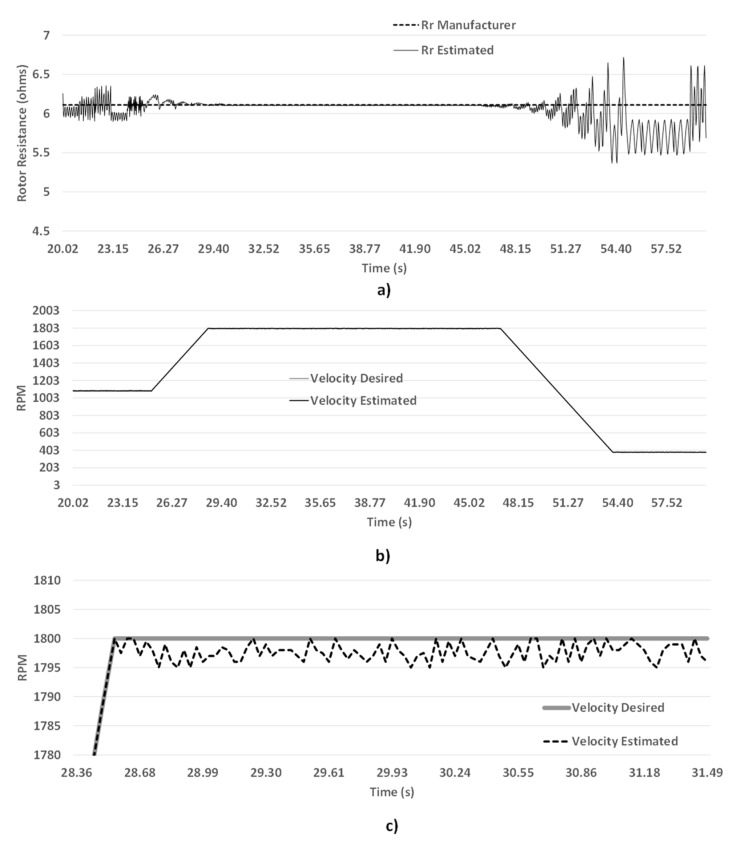
(**a**) Estimated *R_r_* *vs.* Desired *R_r_*; (**b**) Estimated ω_*r*_ *vs.* Desired *ω_r_*; (**c**) Zoom to the velocity.

As it can be observed in Figure 9, ω_*r*_ and *R_r_* value vary at speed changes because the currents and the magnetic fluxes are changed depending on IM electric demand, affecting the controller function. Never the less, the proposed algorithm updates these parameters and introduces them to the FOC scheme and improves driver performance. But, at constant speeds, resistance and speed values keep almost at the same value. Figure 9a shows the *R*_r_ and Figure 9b presents speed estimation respectively, finally, Figure 9c presents a zoom applied to the speed estimation.

## 4. Conclusions

The main contribution of this work is proposing a new kind of estimator for control schemes for neural network and control theory. Along, in this article, the IM speed and rotor resistance estimation was validated and the IM driver performance was improved, due to the inclusion of the CMAC-ADALINE block into the standard FOC scheme. A new hybrid neural network estimator was designed based on two types of neural network structures and resulted as expected.

This estimator adjusts its set of weights in order to update these two values (speed and rotor resistance), which improves FOC algorithm behavior. The implementation of this algorism was easy to program, on a real-time application over a three-phase IM, and good speed and resistance tracking with a minimum error was achieved. As it can be observed, that results are very satisfactory considering the IM was connected to a dynamic load system, which was modified during the test.

The employed kit was only for test and validation of the FOC scheme added with the ANN estimator and it is not for industrial usage yet, a higher power capacity platform must be built to meet the requirements based on the power demand of the application. Future projects stablish the need to develop a more power capacity station and improve of this control scheme to meet industry standards, to command heavier loads and to have the correct accuracy.

## Nomenclature

$V_{ds},V_{qs}$: d-q axes stator voltages
$i_{ds},i_{qs}$: d-q axes stator currents
${𝜙}_{ds},{𝜙}_{qs}$: d-q axes voltage model rotor fluxes in stationary reference frame
$\lambda_{ds},\lambda_{qs}$: d-q axes current model rotor fluxes in stationary reference frame
$\sigma =1-\frac{L_{m}^{2}}{L_{s}L_{r}}$: Leakage coefficient
$\omega_{r}$: Electrical rotor angular velocity
$T_{r}=\frac{L_{r}}{R_{r}}$: Rotor time constant

## References

[1] Brunner C.U., Waide P., Jakob M. (2013). Harmonized Standards for Motors and Systems Global Progress Report and Outlook. Proceedings of the 7th International Conference EEMODS'11 Energy Efficiency in Motor Driven Systems.

[2] Gómez-Espinosa A., Hernández-Guzman V.M., Bandala-Sanchez M., Jiménez-Hernández H., Rivas-Araiza E.A., Rodríguez-Resendiz J., Herrera-Ruíz G. (2013). A New Adaptive Self-Tuning Fourier Coefficients Algorithm for Periodic Torque Ripple Minimization in Permanent Magnet Synchronous Motors (PMSM). Sensors.

[3] Mouna B.H., Lassaad S. (2010). Neural network speed controller for direct vector control of induction motors. Int. J. Eng. Sci. Technol..

[4] Gutierrez-Villalobos J.M., Rodriguez-Resendiz J., Rivas-Araiza E.A., Mucinob V.H. (2013). A review of parameter estimators and controllers for induction motors based on artificial neural networks. Neurocomputing.

[5] Rubaai A., Kotaru R., Kankam M.D. (2001). Online training of parallel neural network estimators for control of induction motors. IEEE Trans. Ind. Appl..

[6] Ye X., Zhang Q., Zhang T. Speed estimation of induction motor based on neural network. Proceedings of the International Conference on Intelligent Control and Information Processing (ICICIP).

[7] Chen T.-C., Sheu T.-T. (2002). Model reference neural network controller for induction motor speed control. IEEE Trans. Energy Convers..

[8] Korlinchak C., Comanescu M. (2012). Sensorless field orientation of an induction motor drive using a time-varying observer. IET Electr. Power Appl..

[9] Roncero-Sánchez P., Garcia-Cerradab A., Feliu-Batllea V. (2007). Rotor-resistance estimation for induction machines with indirect-field orientation. Control Eng. Pract..

[10] Marei M.I., Shaaban M.F., El-Sattar A.A. (2009). A speed estimation unit for induction motors based on adaptive linear combiner. Energy Convers. Manag..

[11] Tsai C.-H. CMAC-based speed estimator design for induction motor drive. Proceedings of the IEEE International Conference on Systems, Man and Cybernetics (SMC).

[12] Bechouche A., Sediki H., Abdeslam D.O., Haddad S. (2012). A Novel Method for Identifying Parameters of Induction Motors at Standstill Using ADALINE. IEEE Trans. Energy Convers..

[13] Sedikia H., Bechouchea A., Abdeslamb D.O., Haddada S. (2013). ADALINE approach for induction motor mechanical parameters identification. Math. Comput. Simul..

[14] Brandstetter P., Cajka R., Skuta O. Rotor time constant adaptation with ANN application. proceedings of the European Conference on Power Electronics and Applications.

[15] Liu H.-K., Tsai C.-H., Lu H.-C. CMAC neural network application for induction motor drives. Proceedings of the IEEE International Conference on Systems, Man, and Cybernetics (SMC).

